# Historical Perspective and Current Trends in Anticancer Drug Development

**DOI:** 10.3390/cancers16101878

**Published:** 2024-05-15

**Authors:** Katarzyna Gach-Janczak, Joanna Drogosz-Stachowicz, Anna Janecka, Karol Wtorek, Marek Mirowski

**Affiliations:** 1 Department of Biomolecular Chemistry, Medical University of Lodz, Mazowiecka 6/8, 92-215 Lodz, Poland; katarzyna.gach@umed.lodz.pl (K.G.-J.); anna.janecka@umed.lodz.pl (A.J.); karol.wtorek@umed.lodz.pl (K.W.); 2 Clinical Trials Support Unit, Medical University of Lodz, Pomorska 251, 92-215 Lodz, Poland; joanna.drogosz@umed.lodz.pl; 3 Laboratory of Molecular Diagnostics and Pharmacogenomics, Department of Pharmaceutical Biochemistry and Molecular Diagnostics, Medical University of Lodz, Muszynskiego 1, 90-151 Lodz, Poland

**Keywords:** cancer chemotherapy, alkylating agents, intercalating agents, mitotic disruptors, kinase inhibitors

## Abstract

**Simple Summary:**

Cancer is one of the leading causes of death worldwide. Among several therapeutic options available for patients, chemotherapy remains the most often used treatment strategy. Anticancer drugs known today are either substances isolated from plants and their derivatives or completely synthetic compounds. Some of them were discovered by accident, others as a result of long-term research. In this review, we present a brief history of the development of the main groups of anticancer drugs, focusing especially on their mode of action. This article also addresses the major side effects and limitations of currently available anticancer drugs and future perspectives in this area.

**Abstract:**

Cancer is considered one of the leading causes of death in the 21st century. The intensive search for new anticancer drugs has been actively pursued by chemists and pharmacologists for decades, focusing either on the isolation of compounds with cytotoxic properties from plants or on screening thousands of synthetic molecules. Compounds that could potentially become candidates for new anticancer drugs must have the ability to inhibit proliferation and/or induce apoptosis in cancer cells without causing too much damage to normal cells. Some anticancer compounds were discovered by accident, others as a result of long-term research. In this review, we have presented a brief history of the development of the most important groups of anticancer drugs, pointing to the fact that they all have many side effects.

## 1. Introduction

Cancer is one of the leading causes of death in developed and developing countries. According to the analyses using the Global Cancer Observatory 2020 (GLOBOCAN), there were an estimated 19.3 million new cancer cases worldwide in 2020, and almost 10.0 million people died due to this disease [1]. Breast cancer in women and lung, colon, prostate, and stomach cancers were the most common ones [2].

Cancer is a multifactorial disease that mostly affects people over the age of 50. However, over the past decades, a rapid increase in early-onset cancers, defined as cancers diagnosed in adults below 50, has been observed [3,4]. Depending on the type and stage of cancer, patients are treated with traditional therapies, including surgery, chemo-, and radiotherapy, or newer forms of treatment, such as immunotherapy [5,6], targeted [7,8,9], hormonal [10], gene [11], and photodynamic therapy [12]. Chemotherapy is an effective and widespread cancer treatment strategy that uses one or more cytostatic drugs. Chemotherapy is the only approach that is systemic, which means that drugs circulate in the blood, reaching cancer cells in all body tissues. They can destroy both original cancer cells and metastatic ones. Cancer cells typically divide faster than healthy tissue, so cytotoxic compounds that affect cell division have a greater effect on tumors than on healthy cells. There are important exceptions to this generalization—bone marrow tissue, gastrointestinal tissue, and hair follicles also divide much faster than other tissues and are highly susceptible to anticancer drugs, which explains the common side effects of cancer chemotherapy, including the drop in the red blood cell count, nausea and hair loss. Despite these serious toxic effects and the possibility of developing resistance to the drug [13,14], chemotherapy still remains the main treatment option, especially in more advanced cancer stages. Therefore, the efforts of scientists around the world are focused on searching for new candidates for more efficient and better tolerated anticancer drugs. Such candidates can be either substances isolated from plants and their derivatives or completely synthetic compounds.

Here, we describe the most important achievements in the field of anticancer drug discovery and future perspectives in this area. The main focus of this review is to show how the major groups of anticancer drugs were discovered and what the mode of action of compounds in each group is.

## 2. The Beginnings of Chemotherapy

The healing properties of medical plants have been known in traditional medicine for centuries. Natural substances that are present in fruit, vegetables, herbs, and spices have been used in ancient Arabian, Indian, and Chinese cultures as remedies for various ailments including cancer [15].

The history of oncological chemotherapy dates back to the 19th century. In 1861, Robert Bentley documented the local anticancer effects of an extract from the roots of *Podophyllum peltatum*. The active ingredient, podophyllotoxin, isolated 20 years later, was shown to inhibit the formation of the mitotic spindle, thus keeping cancer cells in metaphase [16].

**Figure 1:**
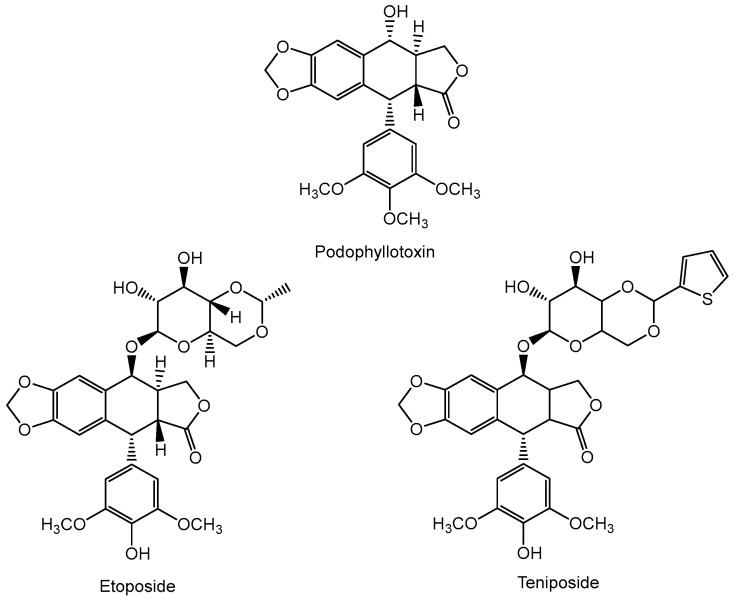
The structure of podophyllotoxin and its synthetic analogs, etoposide and teniposide.

This discovery inspired the design and chemical synthesis of two structural analogs of podophyllotoxin, etoposide and teniposide (Figure 1), which were developed in the late 1960s and early 1970s [17]. Etoposide was approved in 1983 by the US Food and Drug Administration (FDA) for use in anticancer therapy and is still one of the most active drugs in the treatment of testicular cancer, small cell lung cancer, Hodgkin’s lymphoma, non-Hodgkin’s lymphoma, acute myeloid leukemia, ovarian cancer, and gestational trophoblastic neoplasia [18].

The mechanism of action of etoposide involves the formation of a complex with topoisomerase II [19]. DNA topoisomerases are essential enzymes that regulate the topology of the genetic material by introducing transient breaks in the DNA molecule [20]. Topoisomerase II enables the removal of negative and positive supercoils in DNA by generating transient double-strand breaks in the double helix, followed by relaxation of the DNA structure and reconnection of the broken strands. Etoposide stabilizes the covalent enzyme–DNA complex by forming a cleavage complex, which induces double-strand breaks in the DNA and prevents topoisomerase II from rebinding them. The accumulation of permanent DNA breaks triggers recombination/repair pathways, mutagenesis, and chromosomal translocations. If breaks in DNA exceed the cell’s repair capacity, they can initiate pathways that lead to cell death [21]. Thus, etoposide converts topoisomerase II from an essential enzyme into a potent cellular toxin that fragments the genome. Cancer cells, which divide faster and require greater topoisomerase II activity, are more sensitive to etoposide than normal cells.

## 3. Mustard Gases and Their Analogs

**Figure 2:**
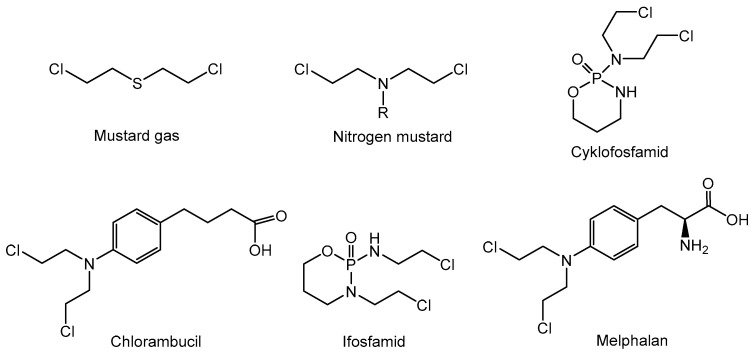
The structure of bis(2-chloroethyl)sulfide (mustard gas) and nitrogen analogs used as anticancer drugs.

The history of systematic clinical chemotherapy dates back to the period after World War I, when the group of Edward B. Krumbhaar from the Department of Research Medicine at the University of Pennsylvania conducted research on the war gasses, specifically on bis(2-chloroethyl)sulfide called mustard gas, which caused the deaths of hundreds of soldiers [22]. During the autopsy of their bodies, it was observed that the soldiers suffered from, among others, a significant reduction in the number of leukocytes. Simultaneous studies conducted at the US Chemical Warfare Service involving rabbits, injected with close to lethal doses of bis(2-dichloroethyl)sulfide, showed a marked decrease in the number of leukocytes in the blood and damage to the bone marrow immediately after compound administration, attributing leukopenia to this compound [23]. Recognition followed that less toxic analogs might show potential in the field of cancer therapy, which led to the synthesis of nitrogen derivatives of mustard gas. Currently, several structural analogs of mustard gas are used in oncological therapy, including cyclophosphamide, chlorambucil, ifosfamide, and melphalan [24,25] (Figure 2).

**Figure 3:**
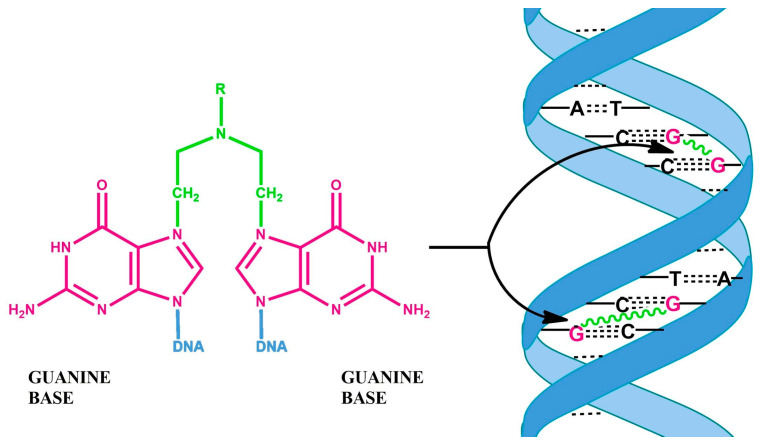
Bifunctional alkylating agents can cause irreversible intra- and interchain links.

Active metabolites of these compounds attach an alkyl group to DNA guanine, resulting in the formation of irreversible intra- and interchain DNA cross-links, which inhibit DNA replication and eventually lead to apoptosis (Figure 3) [26,27,28,29].

## 4. Folic Acid Antagonist

**Figure 4:**
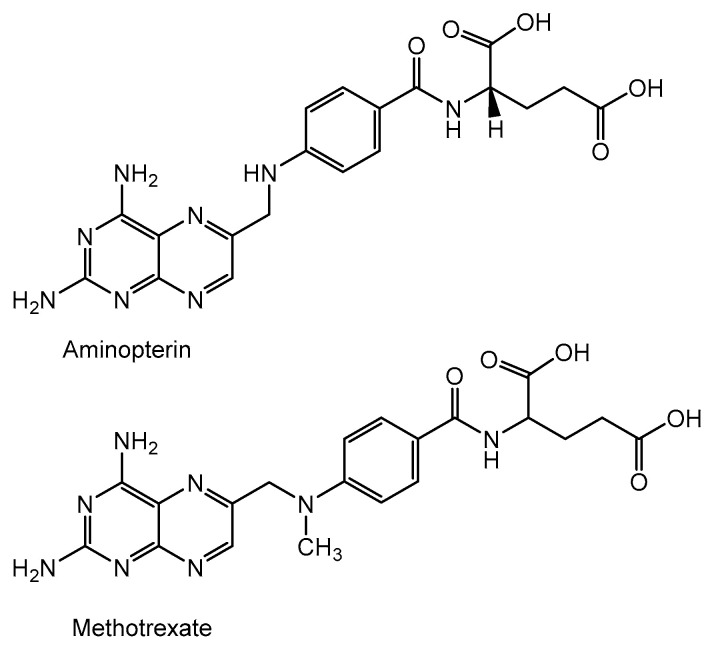
Structural formulas of folic acid antagonists: aminopterin and methotrexate.

The nutritional research performed before and during World War II led to the discovery of a factor, present in green leafy vegetables, that is important for the proper functioning of the bone marrow. This factor was then crystallized, chemically identified, synthesized between 1943 and 1945, and named folic acid [30,31]. A pathologist, Sidney Farber, noted that folic acid stimulates the growth of leukemia cells and accelerates the progression of the disease. He hypothesized that antagonistic folic acid analogs could inhibit or stop cancer cell proliferation. In 1948, he published his research showing that a number of folic acid antagonists, including 4-aminopteryloylglutamic acid (aminopterin), caused transient remissions in children with acute undifferentiated leukemia [32,33]. These observations led to the development of other chemotherapeutics. The less toxic and equally effective methotrexate (Figure 4) replaced aminopterin in clinical practice and is now used as the first-choice drug for the treatment of leukemias and lymphomas [34].

The mechanism of action of methotrexate involves the indirect inhibition of cell division by blocking enzymes related to folic acid, mainly dihydrofolate reductase, which catalyzes the conversion of dihydrofolate to tetrahydrofolate [35]. Tetrahydrofolate is an important coenzyme in several transmethylation reactions in the pyrimidine and purine nucleotide synthesis pathways essential for the synthesis, repair, and replication of DNA strands. The inhibition of intracellular tetrahydrofolate production by methotrexate causes the inhibition of DNA synthesis and also indirectly causes RNA and protein synthesis, the disruption of cell proliferation, and their metabolic balance [36]. Methotrexate acts specifically on proliferating cells, mainly in the S phase of the cell cycle, which makes it more toxic to rapidly dividing cells that replicate their DNA more frequently [37].

Further research concerned a new generation of folic acid antagonists, which were designed to affect key folic acid-dependent enzymes and inhibit their action, leading to the inhibition of nucleotide biosynthesis and, consequently, to cell death [38]. Such new antagonists include pemetrexed indicated as first-line treatment for patients with non-small cell lung cancer or malignant pleural mesothelioma [39,40,41], raltitrexed for advanced colorectal cancer [39,42] and trimetrexed used for the treatment of moderate-to-severe Pneumocystis carinii pneumonia (PCP) [43].

The mechanism of action of pemetrexed is the inhibition of at least three enzymes: thymidylate synthase, dihydrofolate reductase, and glycinamide ribonucleotide formyltransferase, which are involved in folic acid metabolism and the synthesis of purines and pyrimidines. The inhibition of folate metabolism leads to the inhibition of cell growth. In cells, pemetrexed is very quickly converted to active polyglutamate derivatives, which are accumulated and maintained in the cell for a long time, causing prolonged and strong inhibition of thymidylate synthetase. The accumulation of polyglutamates occurs mainly in cancer cells and, to a lesser extent, in normal body tissues, such as progenitor cells of the bone marrow or gastrointestinal tract [44,45].

Raltitrexed and trimetrexate have similar effects. Raltitrexed inhibits thymidylate synthase directly and specifically, without requiring any modulating agent. Raltitrexed is transported into cells via the folate transporter and is then extensively converted by the enzyme folyl-polyglutamate synthetase into polyglutamates, which are retained in the cells. Similarly to pemetrexed, polyglutamation, due to raltitrexed conversion, increases the duration of thymidylate synthase inhibition [46]. Trimetrexate, in turn, is a strong inhibitor of dihydrofolate reductase, which reduces the level of tetrahydrofolate in the cell and leads to disruption of thymidylate biosynthesis. Additionally, this compound indirectly inhibits purine biosynthesis. Both of these effects may result in the inhibition of DNA and RNA synthesis [47]. Trimetrexate has also been shown to act mainly in the S phase of the cell cycle [43].

## 5. Antimetabolites

**Figure 5:**
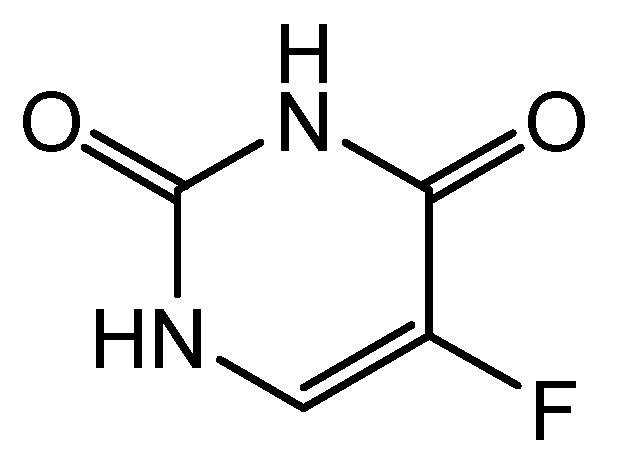
Structure of 5-fluorouracil.

It was not until the mid-1950s that a drug targeting non-hematologic cancers was developed. In 1957, based on the observation that cancer tissues consume uracil faster than normal tissues, 5-fluorouracil (5-FU) (Figure 5) was identified as an effective chemotherapeutic agent [48]. It was found that this agent has a broad spectrum of activity against many solid tumors. Up to now, 5-FU remains the basis of treatment for colorectal cancer, other cancers of the digestive tract (rectum, pancreas, esophagus, stomach, and endometrium), as well as cancers arising in other organs (i.e., breast, cervical, and head and neck cancers) [49]. According to the World Health Organization, 5-FU is one of the most important drugs used in medicine [50]. The cytotoxic action of this chemotherapeutic agent is multidirectional, and it includes the incorporation of its metabolites into RNA and DNA and inhibition of thymidylate synthase. Since thymidine is essential for DNA replication and repair, the inhibition of thymidylate synthase results in abnormally low dTTP levels and a massive increase in dUTP levels, which is responsible for DNA replication arrest. Moreover, 5-FU can also be incorporated into the structure of RNA, preventing its proper functioning. Therefore, the disruption of DNA and RNA synthesis by 5-FU leads to the inhibition of cell growth and directs the cell towards apoptosis [51,52].

In retrospect, 5-FU can be considered the first example of a targeted therapy that has now become the subject of great attention in anticancer drug development, although in this case, the target was a biochemical pathway rather than a specific molecule.

## 6. Anticancer Antibiotics

**Figure 6:**
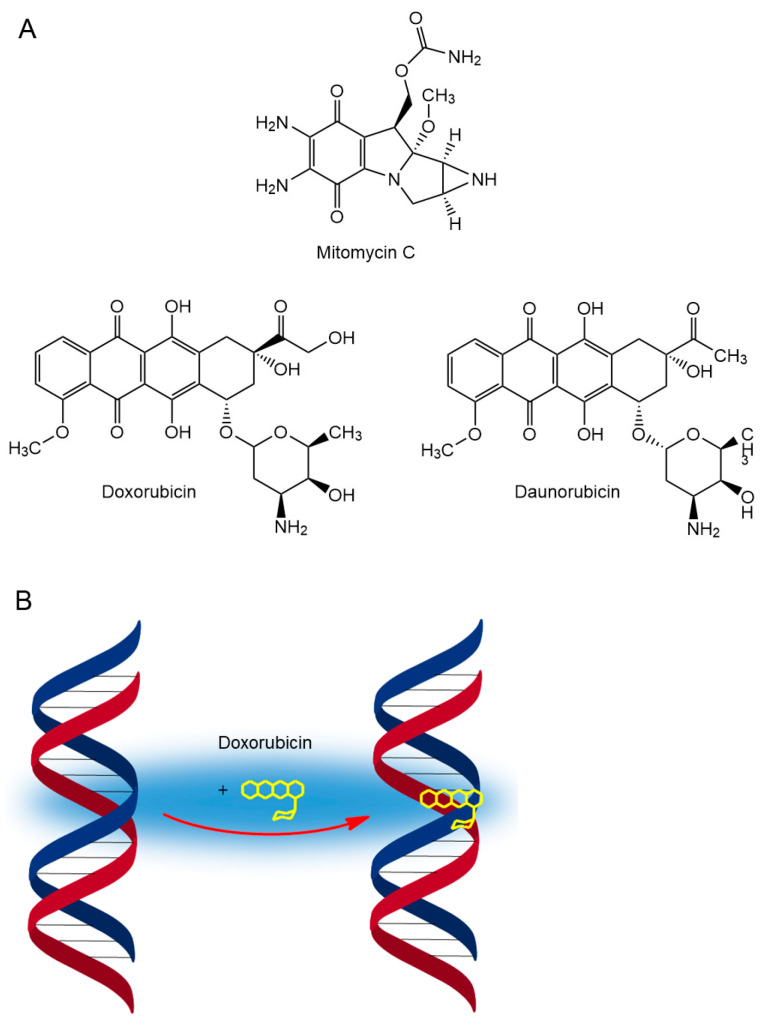
(**A**) Structure of mitomycin C and anthracycline antibiotics: doxorubicin and daunorubicin. (**B**) Intercalation of anthracyclines into DNA.

Fermentation products are an important source of anticancer agents. Mitomycins are a group of potent antibiotics produced by the microorganism *Streptomyces caespitosus*, discovered in Japan in the 1950s. The prototype and the best-studied representative of this group is mitomycin C (Figure 6), showing a broad spectrum of anticancer activity [53]. Subsequent work at Bristol Myers (later Bristol Myers Squibb) resulted in many analogs, but the parent structure is still used in combination chemotherapy with other agents for advanced gastric, breast, pancreatic, and non-small cell lung cancers [54].

Mitomycin is activated in vivo into a biologically active form by reduction. In its original structure, the compound is neutral towards nucleophiles, but after a cascade of reactions initiated by the reduction of the quinone ring, it transforms into an extremely reactive bis-electrophile. The activation of mitomycin generates reactive intermediates that are able to alkylate nucleophiles present in the cell, in particular DNA, forming four monoadducts and two cross-links. The formation of interstrand DNA–DNA cross-links is considered to be the main cause of the cytotoxic effect of this compound [55].

Other important fermentation products include the structurally related anthracycline antibiotics, daunorubicin and doxorubicin (Figure 6), both isolated from *Streptomyces peucetius* in the early 1960s [56]. Anthracyclines, which are flat structures, exert their antineoplastic activity through intercalation into DNA and disruption of topoisomerase II-mediated DNA repair [57], which eventually leads to DNA damage and the generation of reactive oxygen species (ROS), mediating cellular damage [58,59]. More recently, it has been discovered that doxorubicin’s sugar moiety destabilizes the nucleosome by competing for space with histones. As a result, histones are evicted from chromatin, leading to chromatin damage [60].

Unfortunately, doxorubicin causes serious side effects, and the heart is the preferential target of this drug toxicity. Among the mechanisms involved in doxorubicin-induced cardiotoxicity, the main one is oxidative stress, resulting from ROS formation [60,61]. In addition to treatment-limiting cardiotoxicity, therapy-related malignancies and gonadotoxicity are also associated with anthracycline use [60]. Although several hundred synthetic analogs of natural anthracycline antibiotics have been evaluated in subsequent years, only a handful show any additional benefit over the parent structures, which are still used in the treatment of acute lymphocytic and myelocytic leukemia, despite their cardiotoxic side effects [60,62].

## 7. Anticancer Compounds of Plant Origin

**Figure 7:**
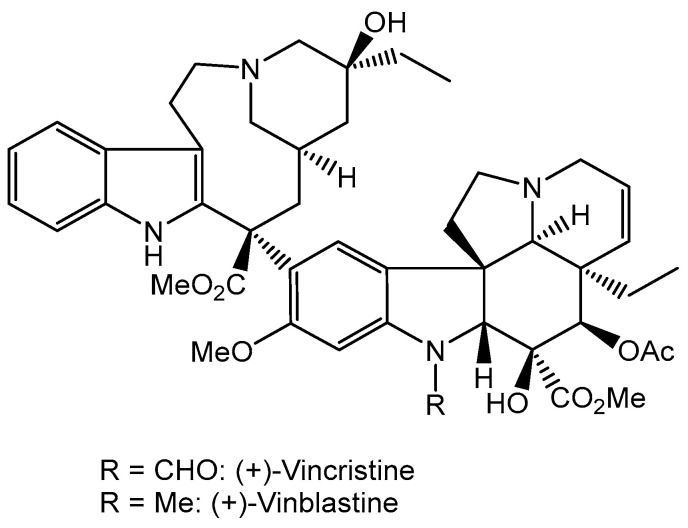
The structure of vinca alkaloids.

More than half of the anticancer drugs currently in clinical use are natural products or their derivatives, and many of them are of plant origin [63]. Extracts from the Madagascar periwinkle (*Catharanthus roseus*), which had been used in folk medicine to treat diabetes, were found to reduce white blood cell count and cause bone marrow depression in rats. Further research led to the isolation of vinblastine and vincristine (collectively named vinca alkaloids) (Figure 7), as active substances [64]. Newer semi-synthetic analogs of these agents are vinorelbine and vindesine [65]. These drugs are used primarily in combination with other chemotherapy agents to treat a variety of cancers, including leukemias, lymphomas, advanced testicular cancer, breast and lung cancers, and Kaposi’s sarcoma [66,67].

Vinca alkaloids alter microtubule dynamics, leading to cell growth arrest and apoptosis. Microtubules are components of the cytoskeleton of the mitotic spindle, which is responsible for chromosome separation during mitosis and meiosis [68]. Microtubules, made of smaller subunits, tubulin molecules, are necessary for maintaining proper cellular structure, transport, and many other cellular processes. Vinblastine and vincristine bind to tubulin, preventing its polymerization into microtubules, which leads to the inhibition of cell division in metaphase. The vital functions of such cells are disturbed, which consequently leads to their apoptosis. The anticancer effect of vinca alkaloids may also be due to the inhibition of DNA repair and RNA synthesis by inhibiting the enzyme DNA-dependent RNA polymerase [67].

**Figure 8:**
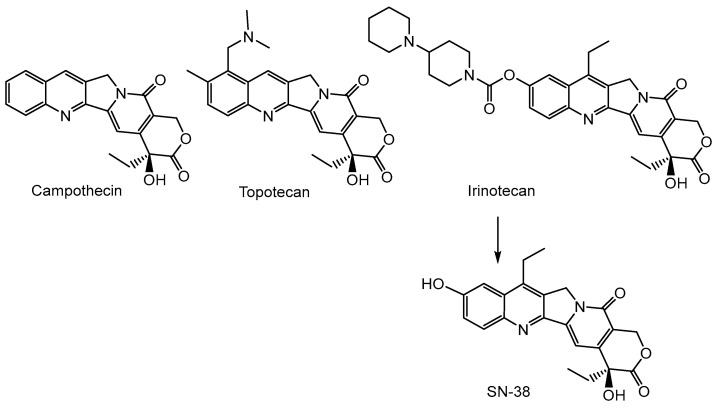
DNA-topoisomerase I inhibitors.

The discovery of vinblastine and vincristine initiated an extensive search by the United States National Cancer Institute (NCI) for other anticancer agents present in plants [63]. In the 1950s, the program began to collect plant samples to search for phytosteroids that could be precursors of cortisone. Thousands of plants, especially rare species, were collected, and their extracts, in addition to research on phytosteroids, were also tested for anticancer activity. Of the initial 1000 plant extracts tested, the extract from *Camptotheca acuminata*, a plant native to China, was the only one with high anticancer activity. In the 1960s, camptothecin (Figure 8) was isolated from *C. acuminata* extracts and described as a “novel alkaloid inhibitor of leukemia and cancer” [69]. However, camptothecin had poor stability and solubility, which was observed early on in the discovery stage. Moreover, unexpected adverse effects such as myelosuppression, vomiting, and diarrhea resulted in the suspension of clinical trials in the early 1970s. Camptothecin gained attention again in the late 1980s, when DNA topoisomerase I was identified as its molecular target and possibly a single point of biological activity [70,71,72]. Various derivatives of camptothecin have been developed to overcome the solubility and stability problems, but only two of them, irinotecan and topotecan (Figure 8), have been approved for clinical practice and are currently used in the treatment of ovarian and colorectal cancer [73]. In contrast to the structurally related topotecan, irinotecan is a prodrug that requires bioactivation to give SN-38 [74].

Topotecan and irinotecan, like camptothecin, interact with topoisomerase I, an enzyme that modulates the topological structure of DNA by inducing transient DNA breaks in one of the two strands of the double-stranded DNA [75]. These single-strand breaks help remove excess positive and negative DNA supercoils that occur during DNA replication and transcription [76]. Under normal conditions, after DNA has been relaxed, topoisomerase I rejoins the broken strand. The interaction between camptothecin and its derivatives and the enzyme results in the formation of a topoisomerase I complex with DNA, which prevents the religation of a single DNA strand. The formation of a three-component complex composed of DNA, topoisomerase I, and a camptothecin analog disrupts the movement of the replication fork, which causes replication to stop, creates double-strand breaks in the newly formed DNA, and consequently leads to cell death. Unlike topoisomerase II, cellular levels of topoisomerase I are independent of the cell cycle phase in normal tissues, so topoisomerase I activity is only slightly increased in dividing cells. Higher constitutive activity of this enzyme can be detected in several cancer tissues, e.g., colorectal adenocarcinoma, which enables the effective action of topoisomerase I inhibitors [77,78].

**Figure 9:**
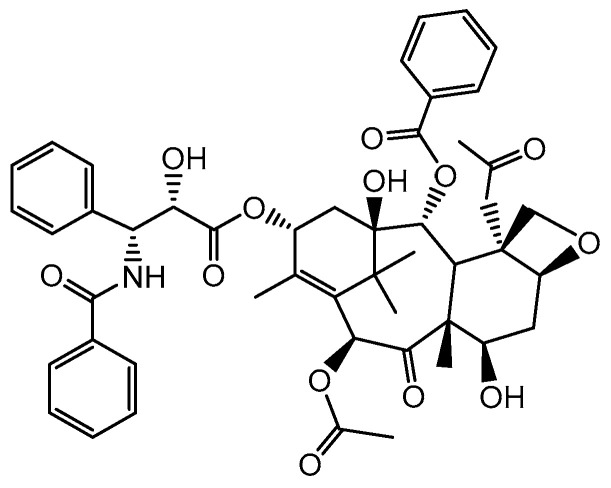
Structure of paclitaxel.

In 1955, the American NCI established a screening program to search for compounds with anticancer activity in the plant world. Thousands of collected leaf, bark, and flower samples were tested for their effectiveness against a selected panel of cultured cancer cell lines. The result of these studies was the isolation of a natural product from the bark of the Pacific yew, *Taxus brevifolia*, which was named paclitaxel (Taxol) (Figure 9) [79,80].

**Figure 10:**
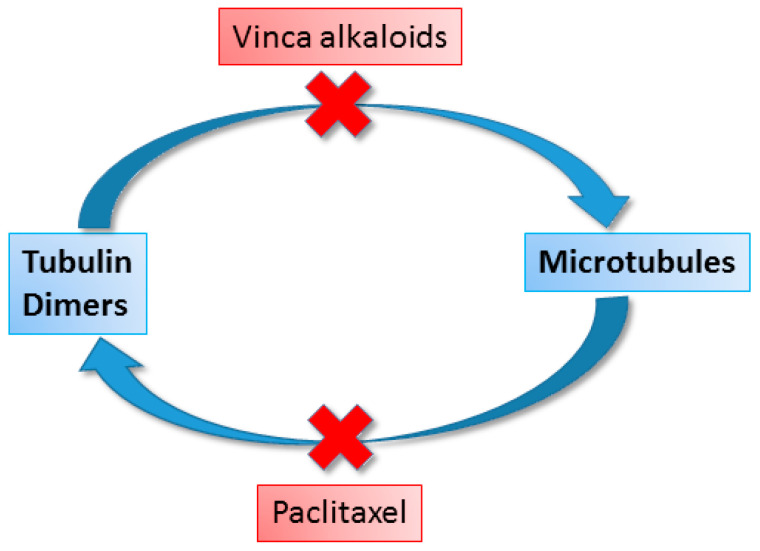
Mechanism of action of vinca alkaloids and paclitaxel.

The mechanism of its cytotoxic effect was discovered in 1979, when it was found that paclitaxel interacts with cell microtubules [81,82]. Paclitaxel works by selectively binding to the β-subunit of the tubulin protein, promoting its polymerization and assembly, which stabilizes microtubules and leads to the accumulation of microtubule bundles in cells [83]. As a result, a dysfunctional mitotic spindle is formed, which causes complete arrest of mitosis in the G2/M phase and eventually leads to cell death by apoptosis. Moreover, paclitaxel reduces tumor angiogenesis and induces the expression of genes and cytokines, leading to the inhibition of tumor cell growth. A combination of both antiproliferative and cytotoxic properties contributes to the anticancer effectiveness of paclitaxel [84]. Paclitaxel has the opposite effect on microtubules to vinca alkaloids, preventing their depolymerization (Figure 10) [85].

Initial concerns regarding the use of paclitaxel in clinical practice focused on the availability of the raw material [86]. It was calculated that the bark of 3–6 whole 100-year-old trees would be needed to provide a sufficient amount of paclitaxel (2 g) for clinical trials for just one person. For this reason, a semi-synthetic approach was developed in which the main skeleton, available from the needles of the English yew *Taxus baccata*, was combined with a synthetic “side chain” [80]. Total synthesis of paclitaxel is also possible, but this process requires many steps and expensive chemical reagents, takes place under reaction conditions that are extremely difficult to control, and the yield is too low to meet the requirements of industrial production [87]. Plant cell cultures are a more promising and sustainable way to produce paclitaxel, but production costs are high and yields are unpredictable [88]. Paclitaxel is currently produced mainly by semisynthesis, which includes the modification of paclitaxel precursors, baccatin III and 10-deacetylbaccatin III, isolated from *Taxus brevifolia* cell cultures [89]. In late 1992, paclitaxel, under the brand name Taxol, was approved for the treatment of ovarian cancer, in 1994 for the treatment of breast cancer, and later for the treatment of non-small-cell lung cancer and Kaposi’s sarcoma, becoming one of the most promising anticancer agents that have appeared since World War II [80].

## 8. Accidental Discoveries of Anticancer Drugs

**Figure 11:**
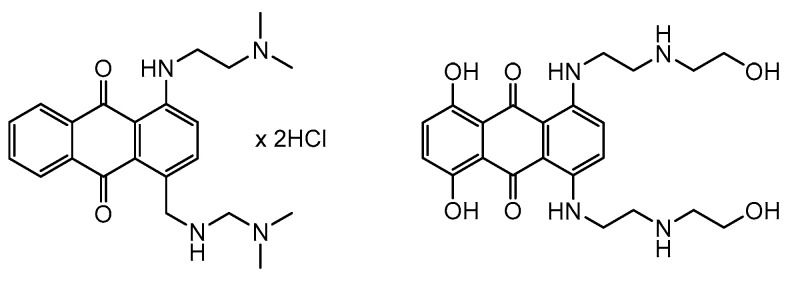
Structure of initial compound CL 55,343 and mitoxantrone.

Serendipity often plays an important role in the discovery of drugs. A classic example is the drug mitoxantrone. The Allied Chemical Company produced a new anthracenedione (designated CL 55,343) to be used as ink and submitted this compound to the NCI for routine screening tests, in which it showed an unexpectedly high degree of anticancer activity. The NCI had previously evaluated many synthetic anthracenediones in their program but none of them showed any antineoplastic activity. The NCI obtained permission to conduct extensive research on the promising new compound and then contracted with the Midwest Research Institute to synthesize its analogs. Of the 47 new compounds synthesized, none showed improved activity. This failure led scientists to compare the structure of the original compound with that of the known anticancer agent, doxorubicin. Structural modifications were made to the original compound using doxorubicin as a model, and the new derivative, 1,4-dihydroxy-5,8-bis{[2-[(2-hydroxyethyl)amino]ethyl]amino}-9,10-anthraquinone (DHAQ), was developed. DHAQ showed ten times higher anticancer activity than the original compound and was eventually approved as an anticancer drug under the name mitoxantrone (Figure 11) [90].

Mitoxantrone is currently indicated for use in the treatment of breast and prostate cancers, non-Hodgkin’s lymphoma, acute myeloid, and nonlymphocytic and lymphoblastic leukemia. Mitoxantrone is a strong poison of topoisomerase II, but it is now clear that the drug interacts with a much broader range of biological macromolecules, both covalently and noncovalently. Cancer cells are sensitive to mitoxantrone treatment in all phases of the cell cycle, but cells in the late S phase are particularly susceptible to the cytotoxic effects of this compound [91].

**Figure 12:**
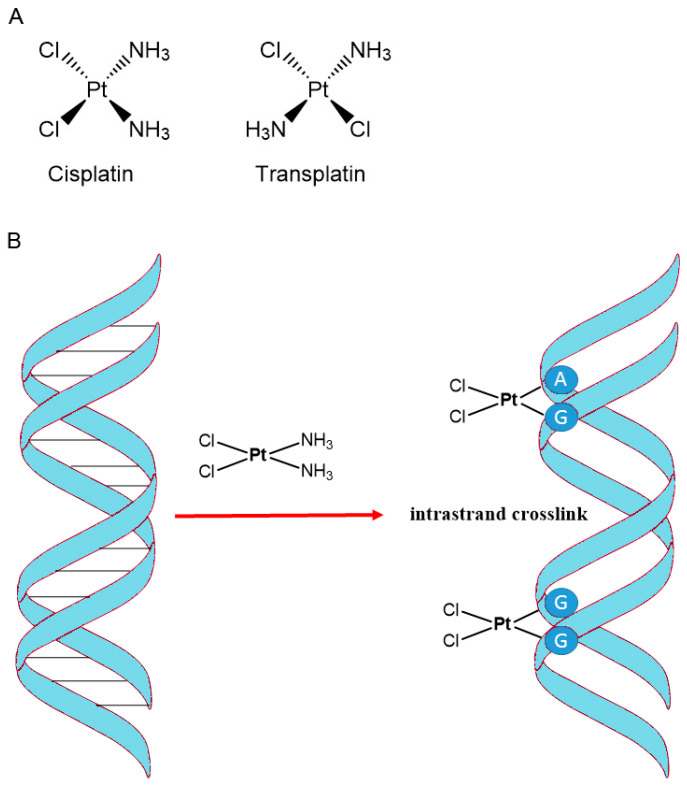
(**A**) Structure of cis- and transplatin. (**B**) Mode of action of cisplatin.

The use of complexes of platinum, palladium, and other noble metals in the treatment of cancer has also aroused considerable interest. One of the most effective compounds from this group is cisplatin (Figure 12) [92]. Cisplatin was discovered in 1845, but its biological properties were not known until 1965, when biophysicist Barnett Rosenberg from Michigan State University discovered its ability to inhibit cell division. Rosenberg studied the effect of an electric field on the growth of *Escherichia coli* and noticed that bacteria stopped dividing. In the experiment, he used platinum electrodes and a solution containing ammonium chloride and *E. coli*. As soon as the current started, the bacterial cells stopped dividing, and when the power was cut off, the cells were dividing again. It was initially thought that the electric field controlled cell division. However, it was later realized that cisplatin, a complex of platinum from the electrodes formed with ammonium chloride, was responsible for this effect. Rosenberg hypothesized that if cisplatin could inhibit bacterial cell division, it could also stop the growth of cancer cells. This assumption turned out to be correct and led to the introduction of cisplatin into anticancer therapy [93,94]. Cisplatin exhibits anticancer activity through many mechanisms, but the most accepted one involves the generation of DNA damage [95,96]. Cisplatin enters the cell via both passive diffusion and active transport [97]. In the bloodstream, where chloride ion concentrations are high (~100 mM) cisplatin maintains its neutral state. In the cell, low chloride ion concentration (~4–12 mM) causes cisplatin to undergo aquation and a chloride is displaced by a water molecule. This is a key step, as the monochloride form is a potent electrophile that rapidly reacts with nucleophiles such as DNA, binding to the nitrogen in the N7 position on purine bases with the loss of the water molecule. The remaining chloride is then subsequently aquated, allowing the cisplatin to cross-link to another purine residue, causing intrastrand or interstrand cross-links, which leads to the inhibition of DNA synthesis and ultimately to apoptosis [98]. Due to its different geometry, transplatin cannot form adducts with DNA and is not used as an anticancer drug [99].

**Figure 13:**
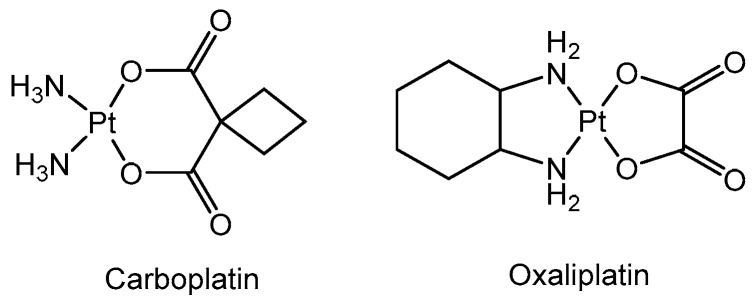
Structure of cisplatin analogs.

Cisplatin is now widely used in the treatment of various solid cancers, especially testicular, ovarian, bladder, head and neck, and lung cancer [100]. However, there are two major concerns associated with cisplatin administration, which are its highly toxic nature, resulting in hearing loss, hemolysis, and nephrotoxicity, and its relatively rapid development of resistance [100]. Out of thousands of cisplatin analogs, two, carboplatin and oxaliplatin (Figure 13), provided a definite advantage over cisplatin and were FDA-approved as anticancer agents [101]. Other more restricted platinum (II) agents are locally used, e.g., nedaplatin (Japan), lobaplatin (China), and heptaplatin (Republic of Korea) [102].

## 9. Tyrosine Kinases Inhibitors

The discovery of imatinib initiated the era of targeted drugs that are much more selective than older generations of cytotoxic agents and have lower side effect profiles. Imatinib was invented in the late 1990s by a biochemist named Nicholas Lyndon, then working for Ciba-Geigy (now Novartis) [103,104]. The first clinical trial of imatinib took place in 1998, and the drug received FDA approval in May 2001. Imatinib (also known as “Gleevec” or “Glivec”) is a tyrosine kinase inhibitor and was called the “magic bullet” when it revolutionized the treatment of chronic myeloid leukemia (CML) in 2001 [105,106]. The disease is caused by a genetic change known as the Philadelphia chromosome or the bcr-abl gene, which produces hyperactive BCR/ABL proteins [107,108].

**Figure 14:**
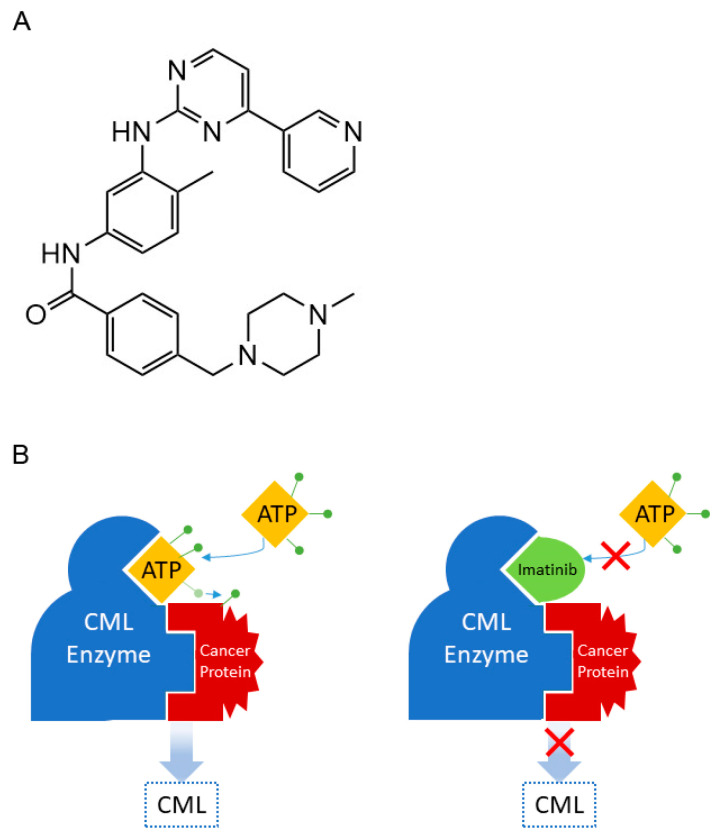
(**A**) Structure of imatinib. (**B**) Mode of action of imatinib.

Chemical libraries had been screened in search of a drug that would inhibit that protein. 2-Phenylaminopyrimidine was identified and used as a lead compound for further modifications. The introduction of methyl and benzamide groups enhanced binding properties and resulted in imatinib (Figure 14A) [109]. Imatinib directly inhibits the constitutively active tyrosine kinase. Tyrosine kinases are important mediators of the signaling cascade, playing key roles in various biological processes such as growth, differentiation, metabolism, and apoptosis in response to external and internal stimuli. Their inhibition ultimately leads to “switching off” the signaling pathways that promote the development of leukemia [110,111]. The active sites of tyrosine kinases each have a binding site for ATP. The enzymatic activity catalyzed by a tyrosine kinase is the transfer of the terminal phosphate from ATP to tyrosine residues on its substrates, a process known as phosphorylation. Imatinib works by binding close to the ATP binding site of BCR-ABL protein, locking it in a closed or self-inhibited conformation, and therefore semi-competitively inhibiting the enzyme activity of the protein [104] (Figure 14B).

Over the last 20 years, more than 40 new tyrosine kinase inhibitors have been introduced into treatment, changing oncological and hematological practice. However, with rare exceptions, such as some cases of chronic myeloid leukemia, patients cannot currently be cured using tyrosine kinase inhibitors as a single drug therapy. Problems related to the development of resistance and toxicity, leading to dose reduction or treatment interruption, are the main challenges associated with their use in cancer patients [105].

## 10. Drugs Targeting Cancer Stem Cells

**Figure 15:**
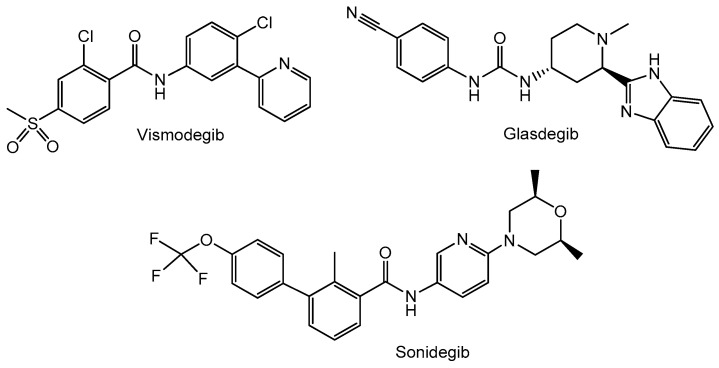
Structure of drugs targeting cancer stem cells.

Currently, the presence of cancer stem cells (CSCs) is believed to be the explanation for the failure of traditional cancer treatments and the main cause of chemo- and radiotherapy resistance [112]. CSCs are a subset of tumor cells with the exclusive ability to self-renew, maintaining tumor growth [113]. In recent decades, the existence of CSCs has been confirmed in many malignancies. It has been discovered that CSCs typically demonstrate persistent activation of one or more highly conserved signal transduction pathways involved in development and tissue homeostasis, including the Wnt, Hedgehog, and Notch pathways [114]. Thus, new treatment strategies targeting these pathways to control stem-cell replication, survival, and differentiation have been under development. Many chemical agents of different classes targeting the pathways affecting CSCs have entered clinical trials, and some inhibitors, such as vismodegib, sonidegib, and glasdegib (Figure 15), have been approved by the FDA as anticancer agents. Vismodegib is a first-in-class small molecule that selectively inhibits the Hedgehog signaling pathway. It binds and inhibits a critical signal transducing component of the Hedgehog signaling, Smoothened (Smo), overexpressed in many types of cancer. Vismodegib was discovered by high-throughput screening of a small molecule compound library, followed by subsequent optimization through medicinal chemistry [115]. Its development can be attributed to the robust collaboration of three biotechnology/pharmaceutical companies (Genentech, Curis, and Evotec). In 2012 vismodegib was approved by the FDA for the treatment of advanced basal cell carcinoma (BCC), that cannot be managed by surgery or radiotherapy [112,115]. Three years later, sonidegib, the second Smo antagonist, developed by Novartis, was introduced to the market in the U.S. and E.U. for the treatment of advanced BCC [116]. Vismodegib and sonidegib are currently being evaluated in phase I/II clinical trials for the treatment of different types of cancer, e.g., breast, gastrointestinal, pancreatic cancer, and medulloblastoma [112]. Glasdegib, developed by Pfizer, is a Hedgehog pathway inhibitor that was approved and launched in the U.S. in 2018 and then in the E.U. for the oral treatment of newly diagnosed acute myeloid leukemia (AML) in adults over 75. It is also undergoing clinical development for use in other hematological malignancies [117].

Unfortunately, the approved Smo inhibitors, used as single-target agents, develop resistance, which limits their efficacy in cancer treatment. It seems that targeting several pathways simultaneously may be a good solution, and further research on CSC inhibitors is heading in this direction.

## 11. Conclusions

Cancer formation is the result of uncontrolled multiplication of cancer cells or an imbalance between proliferation and programmed cell death, or apoptosis. Compounds that can potentially become candidates for new anticancer drugs must have the ability to inhibit the proliferation and induce apoptosis of cancer cells, without causing excessive damage to normal cells. Apoptosis is a natural metabolic process that is crucial in the regulation of tissue homeostasis. It involves removing worn-out and damaged cells from the body, thus protecting it against the harmful consequences that these cells may cause. The mechanism of action of most anticancer drugs is to induce the process of apoptosis in cancer cells [118,119]. However, in cancer, usually too little apoptosis occurs, resulting in malignant cells that will not die.

Despite many successes, anticancer therapy is still one of the greatest challenges of modern medicine. Drug discovery and development is a long, expensive, and high-risk process that typically takes over 10–15 years, and the average cost of approval of each new drug for clinical use is over USD 1–2 billion [120]. Unfortunately, the development process of nine out of ten drug candidates ends in failure in phase I, II, or III clinical trials or at the registration stage. Moreover, when preclinical drug candidates are also included, the failure rate in the drug discovery and development process exceeds 90%. This situation is most often caused by the lack of clinical effectiveness of the substance, its too high toxicity, or its unfavorable physicochemical properties [121]. Continued efforts by scientists and pharmacologists remain essential to developing new drugs to help fight this deadly disease.

Serendipity is one of many factors that have played an important role in the history of drug discovery. However, it is crucial to note that none of the drugs were developed by luck alone. Even accidental discoveries require intuition, knowledge, experience, and critical thinking [122]. During the drug optimization process, lead compounds are optimized through structure–activity relationship (SAR) testing to achieve high affinity and specificity towards their molecular targets.
